# Evaluation of an ADVanced Organ Support (ADVOS) system in a two-hit porcine model of liver failure plus endotoxemia

**DOI:** 10.1186/s40635-017-0144-3

**Published:** 2017-07-04

**Authors:** Ahmed Al-Chalabi, Edouard Matevossian, Anne von Thaden, Catherine Schreiber, Peter Radermacher, Wolfgang Huber, Aritz Perez Ruiz de Garibay, Bernhard Kreymann

**Affiliations:** 1 Jamaica Hospital Medical Center, Phase II Building, 8900 Van Wyck Expy Ste 2, Richmond Hill, New York City, NY 11418 USA; 2 Chirurgische Klinik und Poliklinik, Klinikum rechts der Isar, Technische Universität München, Ismaninger Str. 22, 81675 Munich, Germany; 3 German Center for Neurodegenerative Diseases (DZNE) e.V., Lynen-Str. 17, 81377 Munich, Germany; 4 Institute of Medical and Polymer Engineering, Department of Mechanical Engineering, Technische Universität München, Munich, Germany; 5 Hepa Wash GmbH, Agnes-Pockels-Bogen 1, 80992 Munich, Germany; 6 Institut für Anästhesiologische Pathophysiologie und Verfahrensentwicklung, Universitätsklinikum Ulm, Helmholtzstr. 8/1, 89081 Ulm, Germany; 7 II Medizinische Klinik, Klinikum rechts der Isar, Technische Universität München, Ismaninger Str. 22, 81675 Munich, Germany

**Keywords:** Liver failure, Endotoxemia, Sepsis, Animal model, Swine, Cholestasis, Multiple organ failure, Albumin dialysis, Extracorporeal organ support, Survival

## Abstract

**Background:**

Novel extracorporeal procedures are constantly being developed and evaluated for use in patients with sepsis. Preclinical evaluation of such procedures usually requires testing in large animal models. In the present work, the safety and efficacy of a recently developed ADVanced Organ Support (ADVOS) system in a newly developed large animal two-hit model of liver failure combined with endotoxemia were tested.

**Methods:**

After establishing the model in more than 50 animals, a randomized study was performed. An inflammatory cholestatic liver injury was initially provoked in pigs. Three days after surgery, endotoxin was gradually administered during 7½ h. Animals were randomized to receive standard medical treatment either with (ADVOS group, *n* = 5) or without ADVOS (control group, *n* = 5). The ADVOS treatment was started 2½ h after endotoxemia and continued for 7 h. Survival, cardiovascular, respiratory, renal, liver, coagulation, and cerebral parameters were analyzed.

**Results:**

Three days after surgery, cholestatic injury resulted in hyperbilirubinemia [5.0 mg/dl (IQR 4.3–5.9 mg/dl)], hyperammonemia [292 μg/dl (IQR 291–296 μg/dl)], leukocytosis [20.2 10^3^/μl (IQR 17.7–21.8 10^3^/μl)], and hyperfibrinogenemia [713 mg/dl (IQR 654–803 mg/dl)]. After endotoxemia, the ADVOS procedure stabilized cardiovascular, respiratory, and renal parameters and eliminated surrogate markers as bilirubin [2.3 (IQR 2.3–3.0) vs. 5.5 (IQR 4.6–5.6) mg/dl, *p* = 0.001] and creatinine [1.4 (IQR 1.1–1.7) vs. 2.3 (IQR 2.1–3.1) mg/dl, *p* = 0.01]. Mortality: All animals in the ADVOS group survived, while all animals in the control group expired during the 10-h observation period (*p* = 0.002). No adverse events related to the procedure were observed.

**Conclusions:**

The ADVOS procedure showed a promising safety and efficacy profile and improved survival in a sepsis-like animal model with dysfunction of multiple organs. An amelioration of major organ functions (heart and lung) combined with removal of markers for kidney and liver function was observed.

**Electronic supplementary material:**

The online version of this article (doi:10.1186/s40635-017-0144-3) contains supplementary material, which is available to authorized users.

## Background

Multiple organ failure (MOF) is a major contributor to the mortality of patients with sepsis in the intensive care unit (ICU) [1]. The majority of patients staying longer than 3 days in the ICU already have involvement of the respiratory, cardiovascular, or central nervous system upon admission [2, 3]. Moreover, the two main detoxifying organs, i.e., the liver and the kidney, are also impaired in a high number of patients, ranging from 11 to 25% and from 16 to 67%, respectively [2–5]. The diminished detoxifying function of these two organs results in an accumulation of protein-bound and water-soluble metabolic products that favors the perpetuation of organ dysfunction and contributes to the rapid dysfunction of multiple organs due to the increase of the toxic burden in the human body [6, 7].

Indeed, MOF results from an “altered organ function in an acutely ill patient such that homeostasis cannot be maintained without intervention” [8]. Bearing this in mind, interrupting this vicious cycle appears to be an essential concept in the treatment of e.g. liver and kidney dysfunction and, consequently, sepsis. As proposed by Ronco and Bellomo, single-organ support may be a simplistic view for the management of ICU patients, suggesting that multi-organ support therapy should represent the most logical future conceptual and practical evolution to achieve the goal of extracorporeal blood purification [9]. The newly introduced ADVanced Organ Support (ADVOS) system (previously known as the Hepa Wash procedure), combining liver and renal support, based on albumin dialysis, has been shown to improve dysfunction of the liver and kidney and the circulatory system and survival in an animal model of acute liver failure [10].

The major cause of MOF is sepsis, which has been recently redefined by the European Society of Intensive Care Medicine as a “life-threatening organ dysfunction due to a dysregulated host response to infection” [11]. The severity of the different organ dysfunctions in sepsis and its correlation to mortality can be estimated by the sepsis-related organ failure assessment (SOFA) score, which covers six different organ systems (and parameters) graded from 0 (no dysfunction) to 4 (severe dysfunction/failure), including the liver (bilirubin) and the kidney (creatinine) [12]. The positive correlation of mortality with the SOFA score ranges from less than 30% of deaths for patients with a SOFA score below 9 to more than 70% for a SOFA score higher than 15 [3, 13].

As already described by Meakins, MOF may occur following the two “hit” model [14], where the first hit (e.g., liver injury) would trigger an enhanced inflammatory response that might be followed by a “second hit” or insult (e.g., a nosocomial infection) [15]. In this regard, bacterial toxins (e.g., lipopolysaccharides also called endotoxins) play a major role in the cascade of events occurring in sepsis [16–18]. Animal models involving sepsis and liver injury have contributed to our understanding of many of the underlying pathophysiological pathways. These models are, however, mainly established in small animals (rats or mice) and mostly unavailable for the assessment of safety and efficacy of extracorporeal support systems [19]. To simulate multiple organ dysfunction during sepsis, we developed a two-stage pig model. In order to be validated, the model was required to have increased levels of protein-bound and water-soluble organ dysfunction markers (e.g., bilirubin, creatinine, BUN, lactate) due to a sepsis-like syndrome. To improve the feasibility of the model, death in the control group should occur within 8 h after induction of the second hit (endotoxemia) to allow for termination of the whole procedures within 16 h.

We provoked a cholestatic liver injury by ligation of the main bile ducts. In addition, we established a functional end-to-side portosystemic shunt in order to reduce liver perfusion. The severity and stability of the model and, consequently, the development of the sepsis-like syndrome were further strengthened through the administration of endotoxins.

In the present work, we evaluated the safety and efficacy of the ADVOS system in an animal model with multiple organ involvement and with a high mortality rate when treated with the standard medical treatment. Among others, blood gas, electrolytes, liver and kidney function, and hemostatic, hemodynamic, and cerebral parameters were analyzed. We paid special attention to survival rates, as well as to those parameters related to the SOFA score.

## Methods

### Animals and housing

**Fig. 1:**
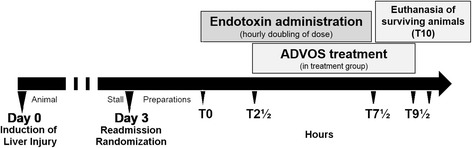
Timeline of the experiments. On day 0, an inflammatory cholestatic liver injury was initially provoked in pigs. Three days after surgery, endotoxins were gradually administered during 7½ h. Animals were randomized to receive standard medical treatment either with (ADVOS group, *n* = 5) or without ADVOS (control group, *n* = 5). The ADVOS treatment was started 2½ h after endotoxemia and continued for 7 h

The study was approved by the ethical committee for animal studies in Bavaria, Germany. Housing and all medical and surgical procedures were performed in the Center for Preclinical Research (ZPF) of the Klinikum rechts der Isar (Munich) in accordance with the national animal protection act (Tierschutzgesetz). German landrace female pigs (~60 kg) were kept in animal housing for about 4–7 days to allow for acclimatization before the surgical procedure. The timeline of the experiments is schematically described in Fig. 1. Every step was performed following carefully prepared standard operating procedures (SOPs) as the study was designed and procedures were put in place to comply with good laboratory practice (GLP) and assure data quality and integrity. The institution has its own policies and procedure in compliance with the local laws and guidelines, but was not GLP certified.

### Surgical procedure

The sepsis-like swine model was developed in two steps: induction of liver injury and further development to multiple organ dysfunction through superimposed endotoxemia. Before the experiments, pigs remained fasting for 12 h with free access to water. In order to prevent gastric and duodenal ulceration, pantoprazol (80 mg) was daily administered from the day of admission of animals in ZPF [20].

On day 0, two cannulas were inserted into the ear veins to establish intravenous access. Intramuscular premedication consisted of ketamine (15 mg/kg), azaperone (2 mg/kg), and atropine (0.5–1 mg/kg). Anesthesia was induced with ketamine (1–2 mg/kg) and 2% propofol (1–2 mg/kg) and was maintained with the latter (60–100 mg, i.v.). The pigs were mechanically ventilated through endotracheal intubation following the recommendations of the Acute Respiratory Distress Syndrome Network [21]. The aim was to keep the arterial oxygen pressure (PaO_2_) around 80 mmHg by adequately adjusting the tidal volume (8 ml/kg). We adjusted the respiratory rate (up to 35) for a better control of the acid-base status. The inspiratory plateau was set at a pressure ≤30 cm H_2_O, which might be exceeded, if necessary, to treat respiratory acidosis (i.e., pH < 7.3). The fraction of inspiratory oxygen (FiO_2_) and positive end-expiratory pressure (PEEP) combinations employed can be found in the supplement (Additional file 1: Table S1). Adequacy of anesthesia was clinically assessed ensuring that animals had sufficient relaxation and analgesia and was adjusted accordingly [22]. Animals received buprenorphine (0.6–1.2 mg/24 h) on day 0 and metamizol (p.o. or i.m. 40 mg/kg) were administered before surgery on day 0 and on days 1 and 2. Intravenous infusions of propofol 2% and remifentanil were used to maintain anesthesia on day 3.

#### Induction of liver injury

Induction of liver injury was performed based on the surgical procedure described by Awad and colleagues [23], with minor modifications [24]. Briefly, on day 0, laparotomy was performed and the bile ducts and portal vein in the hepatoduodenal ligament were exposed in order to ligate the cystic, common hepatic, and the common bile duct (Vicryl^®^ 2/0, Ethicon Inc., Norderstedt, Germany). The latter was ligated twice to ensure complete obstruction of bile flow. Afterwards, the portal vein and inferior (caudal) vena cava were partially clamped before a functional end-to-side portosystemic anastomosis was established. Arterial supply of the liver was not interrupted, and the development of splanchnic congestion was avoided by ensuring an adequate portal flow during partial clamping [24]. Cefuroxime (i.v. 500 mg) was infused during surgery. The animals were returned to their pens where they were clinically observed.

#### Superimposed endotoxemia

On day 3 after induction of liver injury, animals were re-admitted to the operation room and were anesthetized and further challenged with *E. coli* lipopolysaccharide (serotype: B0111:B4, VWR International GmbH, Darmstadt, Germany), starting with a dose of 4 μg/kg/h and continuing with twofold stepwise increments every hour for 7½ h, up to a total dose of 764 μg/kg. The endotoxin was dissolved in saline and administered through an auricular vein. Paracetamol (i.v. 1–2 g) over 15 min was given to all animals participating in the study when endotoxin infusion started. Endotoxins can lead to variable elevation of hypothalamic set point for body temperature with resultant violent shivering and fever of the animals. Paracetamol as antipyretic was given to control these symptoms and avoid differences between groups, which could have led to bias in the final results.

The procedures carried out in this study have been validated in two previous publications where 7 [24] and 14 pigs [10] in each case were employed. In addition, 32 additional animals were necessary in order to set an adequate endotoxin dosing protocol (unpublished observations). Consequently, we have developed a stable swine sepsis-like model that allowed us to evaluate the safety and efficacy of a three-circuit albumin dialysis-based extracorporeal organ support system (ADVOS).

### ADVOS procedure

**Fig. 2:**
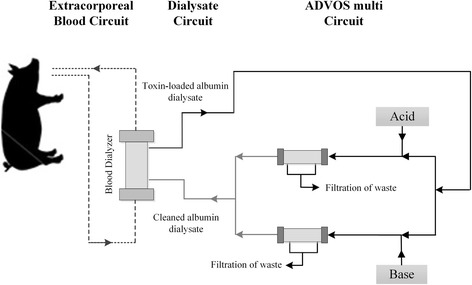
A schematic representation of the laboratory prototype to conduct the ADVOS procedure. Albumin dialysis is performed through a three-circuit system (i.e. blood, dialysate, and ADVOS multi) that allows to eliminate the excess of protein-bound and water-soluble toxins from the patients’ body and to recover albumin from dialysate circuit through a recirculation system

A laboratory prototype (Hepa Wash GmbH, München, Germany) was employed to conduct the ADVOS procedure as already described in [10]. The treatment consists of an albumin dialysis performed through a three-circuit system (i.e. blood, dialysate, and ADVOS multi). The dialysate circuit allows to eliminate the excess of protein-bound and water-soluble toxins from patients’ body (Fig. 2). In the ADVOS multi circuit, toxin-loaded albumin dialysate is divided into two. Before reaching the filters, acid (HCl) or base (NaOH) is added and each part is subjected to a pH and temperature change that favors toxin removal from albumin. The resulting dialysates containing toxin-free albumin join each other in order to reach the desired pH before entering the hemodialyzers.

The treatment was started 2½ h after induction of endotoxemia and continued for 7½ h. Blood circulated between 225 and 250 ml/min through two 1.8 m^2^ surface hemodialyzers (Fresenius Medical Care, Bad Homburg, Germany). Dialysate containing Na^+^, Cl^−^, K^+^, Mg^2+^, HPO_4_^2−^, CO_3_^2−^, glucose, and 3% albumin flowed at 1200 ml/min co-currently to blood. Postdilution (2 l/h) was performed with PrismaSol2^®^ (Gambro Hospal GmbH, Gröbenzell, Germany). Thanks to the recycling circuit, albumin was supplied only at the beginning of the treatment.

The same anticoagulation protocol (with heparin) described previously was employed [10]. However, in order to enable a change to citrate anticoagulation if necessary, the dialysate solution did not contain any calcium. Therefore, external calcium infusions were needed to be administered so that calcium loss was corrected. None of the animals receive citrate anticoagulation throughout the study.

### Experimental design

#### Randomization

Ten pigs were randomly allocated to either control (*n* = 5) or ADVOS group (*n* = 5) following a block randomization with SPSS^®^ for Windows (Additional file 1: Table S2). We performed randomization on day 1 of the experiments.

#### End points of the study

The primary end point of the study was to evaluate the potential survival benefit of the ADVOS procedure in a swine model with a sepsis-like syndrome. Animals were considered dead if cerebral perfusion pressure (CPP) was lower than 5 mmHg for 5 min. Surviving animals were sacrificed with an intravenous lethal dose of pentobarbitone and KCl 10 h after start of endotoxemia (T10). Additionally, the effects of the ADVOS procedure in the course of an endotoxin-induced sepsis-like syndrome were evaluated, paying special attention of those systems involved in the estimation of the SOFA score (coagulation, cardiovascular, cerebral, renal, respiratory, and hepatic systems).

### Monitoring and sample analysis

#### Fluid balance

On day 3, cannulation and adjustment of fluid therapy by the PiCCO system (Pulsion Medical Systems AG, Munich, Germany) were performed, as described previously [10, 24]. A dialysis catheter (13 F high flow two-lumen 20 cm, Achim Schulz-Lauterbach VMP, Iserlohn, Germany) was inserted, placing the tip in the right atrium of the heart. PiCCO parameters were recorded each hour between T0 and T10. The administered fluids were adjusted according to the electrolyte status and included crystalloids like normal saline, dextrose 5–20% with or without KCl and/or bicarbonate. Target for fluid therapy was to keep extravascular lung water index (ELWI) <12 ml/kg and global end-diastolic volume index (GEDI) between 500 and 800 ml/m^2^.

In the case of metabolic acidosis (pH < 7.3), sodium bicarbonate (8.4%) was administered to increase bicarbonate levels (aim 28–30 mmol/l). Infusion fluids were supplemented with potassium (KCl, 20–80 ml, 1 M) or calcium (calcium gluconate 10%, 10–100 ml/h) to prevent hypokalemia (<3 mmol/l), or hypocalcemia (<1.2 mmol/l), respectively. Additionally, potassium levels above 4.8 mmol/l were treated by insulin injections (5–25 IU) in boli with simultaneous adjustment of glucose infusions (5 or 20%, to maintain levels between 110 and 150 mg/dl). Ninety minutes were allowed for hemodynamic parameters to stabilize after completing all surgical procedures.

#### Intracranial pressure measurement

Intracranial pressure (ICP) and temperature were monitored every 15 min between T0 and T10 using an intraparenchymal transducer combined with Datalogger MPR2 logO (Raumedic AG, Münchberg, Germany), as previously described [24].

#### Cardiovascular monitoring

Cardiac rhythm was monitored via a standard lead II electrocardiogram. Hemodynamic and respiratory parameters such as oxygen saturation, arterial blood pressure, end-tidal volume, or heart rate were monitored every 15 min between T0 and T10 using the Compact Critical Care Monitor (Datex-Ohmeda, Helsinki, Finland).

#### Blood sample analysis

Blood samples for biochemical analyses (among others, liver enzymes, creatinine, lactate, BUN, and ammonia) were collected on day 3 just after anesthesia and intubation (day 3 pre-endotoxemia), immediately before induction of liver injury (day 0) and endotoxemia, i.e., after completing minor surgical procedures and stabilization period (T0), and every 2 h after endotoxemia (T2, T4, T6, T8, and T10 or prior to death). Samples were sent to the in-house laboratory. Blood gas analysis (including glucose and electrolyte measurement) was performed more frequently (Rapidpoint^®^ 405, Siemens Health Care Diagnostics Inc., Eschborn, Germany) to ensure quick adjustment of glucose and PaO_2_.

### Statistics

The log-rank test was employed to evaluate survival, whereas Student’s *t* test for paired samples was used to compare the pre-endotoxemia parameters between day 0 and day 3. A repeated measures ANOVA was used to evaluate the effects of the ADVOS procedure on the course of endotoxemia and for intergroup comparison (T6). A two-tailed *p* value lower than 0.05 was considered to indicate statistical significance. Data were documented and analyzed using IBM SPSS 19.0 for Windows^®^. If any data, especially at the end of the experiments, were missing due to death of the animal, they were assumed to be equal to the latest measured value in accordance to the last observation carried forward (LOCF) method [25].

## Results

### Animal post-operatory characteristics (day 0 to day 3)

The liver injury induction operation was well tolerated by the animals, and no apparent wound infections were found. On day 3, post-operatively and before endotoxin administration, animals were stable but developed behavioral changes, jaundice, and dark urine. Weight loss was also evident in both study arms. Differences in biochemical values between day 3 (pre-endotoxemia) and day 0 (before operation) revealed hyperbilirubinemia, hyperammonemia, hyperfibrinogenemia, elevated liver enzymes, and an increase of creatinine levels, suggesting a liver and kidney dysfunction (Table 1).

**Table 1:** Laboratory parameters on day 0 (baseline—before operation) and on day 3 before endotoxin administration

Parameter	Baseline (day 0)		Pre-endotoxemia (day 3)	
	Control (*n* = 5)	ADVOS (*n* = 5)	Control (*n* = 5)	ADVOS (*n* = 5)
Weight (kg)	60.0 (57.8–60.9)	58.0 (57.0–60.6)	60.0 (53.8–60.3)	57.0 (56.3–59.7)
Weight loss (kg)	n.a.	n.a.	2.5 (0.6–3.0)	1.0 (0.9–1.0)
Creatinine (mg/dl)	1.1 (1.1–1.4)	1.5 (1.4–1.6)	1.6 (1.4–1.6)*	1.6 (1.6–1.8)
Urea (mg/dl)	13.0 (10.0–17.0)	12.0 (10.0–12.0)	14.0 (13.0–15.0)	11.0 (7.0–11.0)
Alkaline phosphatase (U/l)	130 (122–151)	154 (152–160)	239 (208–262)*	261 (222–264)*
Total bilirubin (mg/dl)	0.1 (0.1–0.2)	0.2 (0.2–0.2)	5.3 (4.5–5.7)*	4.7 (4.2–5.9)*
GPT (U/l)	34 (28–39)	32 (30–38)	53 (38–59)*	47 (39–48)*
GOT (U/l)	27 (20–31)	31 (28–33)	106 (79–111)*	62 (54–64)*
LDH (U/l)	491 (405–514)	507 (459–535)	779 (757–855)*	629 (608–679)*
Total protein (g/dl)	5.3 (5.2–5.5)	5.8 (5.6–5.8)	5.9 (5.5–6.0)	5.7 (5.6–5.7)
Total calcium (mg/dl)	2.45 (2.38–2.51)	2.49 (2.47–2.51)	2.51 (2.50–2.56)	2.64 (2.57–2.69)
Phosphate (mg/dl)	9.5 (9.0–10–0)	9.0 (8.9–9.2)	8.4 (8.0–8.9)	7.7 (7.5–8.4)
Magnesium (mmol/l)	0.91 (0.85–0.92)	0.88 (0.85–0.89)	0.89 (0.86–0.95)	0.84 (0.81–0.89)
Lactate (mmol/l)	1.3 (1.2–2.3)	2.9 (1.3–2.9)	1.6 (1.5–2.2)	1.4 (1.2–1.6)
Ammonia (μg/dl)	42.0 (39–52)	53.0 (47.0–54.0)	389 (278–403)*	182 (165–210)*
Osmolality (mosmol/kg)	291 (290–291)	288 (288–290)	294 (291–296)*	292 (291–294)*
Albumin (electrophoresis) (g/dl)	2.9 (2.8–2.9)	3.1 (2.9–3.1)	2.8 (2.7–2.9)	2.7 (2.6–3.0)
Quick value (%)	114 (111–119)	106 (105–111)	95 (89–110)*	86.0 (82–96)*
INR	0.9 (0.9–0.9)	0.9 (0.9–1.0)	1.0 (0.9–1.1)*	1.1 (1.0–1.1)*
Fibrinogen (mg/dl)	400 (377–412)	418 (404–434)	665 (629–813)*	716 (710–794)*
Leukocytes (10^3^/μl)	15.2 (14.2–15.9)	15.4 (12.8–16.5)	17.8 (17.7–19.0)*	21.9 (21.4–23.2)*
Hemoglobin (g/dl)	10.0 (9.4–10.1)	10.5 (10.4–11.0)	10.5 (10.5–11.4)	10.2 (10.0–11.3)
Hematocrit (%)	32.8 (32.7–33.0)	34.4 (34.3–37.2)	36.7 (34.6–38.0)	33.7 (33.3–38.1)
Platelets (10^3^/μl)	399 (335–407)	332 (318–402)	393 (359–437)	383 (378–398)

*n.a.* not applicable, *GPT* glutamate-pyruvate transaminase, *GOT* glutamic oxaloacetic transaminase, *LDH* lactate dehydrogenase, *INR* international normalized ratio

*p < 0.05 vs. baseline

Median (IQR)

After the start of endotoxemia and before treatment (T2, before ADVOS), animals in both groups developed signs of septic shock including capillary leak, hemodynamic circulatory changes, reduction of blood cellular elements, coagulopathy, and respiratory failure. In addition, a fall of blood pH with development of lactic acidosis was observed (Table 2).

**Table 2:** Summary of the recorded and measured parameters during the induction of endotoxemia in the ADVOS and control groups. Median (IQR)

		T0		T2		T4		T6^a^		T8	T10
	Parameter	Control (*n* = 5)	ADVOS (*n* = 5)	Control (*n* = 5)	ADVOS (*n* = 5)	Control (*n* = 5)	ADVOS (*n* = 5)	Control (*n* = 5)	ADVOS (*n* = 5)	ADVOS (*n* = 5)	ADVOS (*n* = 5)
Cardiovascular system	MAP (mmHg)	65 (64–65)	69 (66–78)	59 (56–61)	70 (66–71)	59 (53–60)	62 (59–62)	52 (51–53)	61 (56–66)*	68 (61–76)	66 (61–73)
	CVP (mmHg)	3 (2–3)	3 (3–6)	4 (2–4)	3 (2–8)	6 (4–7)	4 (3–9)	7 (6–8)	5 (4–8)	3 (3–4)	4 (3–6)
	Systole (mmHg)	101 (96–108)	101 (95–105)	103 (102–106)	103 (100–104)	100 (97–111)	95 (92–95)	102 (88–103)	97 (94–98)	109 (101–114)	108 (101–121)
	Diastole (mmHg)	52 (49–61)	57 (50–64)	41 (39–50)	55 (48–55)	41 (40–41)	42 (42–45)	36 (31–41)	44 (37–51)*	43 (42–58)	44 (38–50)
	Heart rate (beats/min)	76 (76–81)	73 (69–79)	115 (110–117)	103 (97–103)	120 (115–129)	99 (98–104)	122 (118–140)	109 (102–114)	118 (117–130)	135 (125–147)
	Cardiac index (l/min/m^2^)	5.4 (5.4–6.1)	4.6 (4.2–4.9)	6.8 (6.1–7.8)	5.2 (4.5–5.9)	5.8 (4.0–6.7)	5.9 (5.1–6.2)	4.9 (3.6–5.5)	6.7 (6.1–7.3)*	7.5 (5.3–7.7)	7.0 (6.6–7.5)
	Temperature (°C)	37.8 (37.1–38.0)	38.1 (37.5–38.3)	38.0 (37.2–38.4)	38.3 (37.9–38.4)	37.7 (37.2–38.4)	37.7 (37.2–38.7)	38.0 (37.9–38.5)	37.9 (37.8–38.5)	38.2 (38.0–38.4)	38.4 (37.9–39.0)
	SVRI (dyn.s.m^2^.cm^−5^)	933 (726–941)	1267 (1242–1340)	618 (597–909)	1186 (1122–1249)	685 (639–1001)	868 (691–1019)	830 (764–982)	681 (670–751)	717 (711–907)	716 (676–770)
	GEDI (ml/m^2^)	767 (641–989)	647 (594–698)	652 (551–775)	621 (553–646)	732 (566–749)	809 (671–859)	703 (674–719)	853 (700–976)	859 (653–870)	688 (659–728)
	ITBI (ml/m^2^)	959 (801–1236)	809 (742–872)	815 (689–968)	776 (691–808)	915 (707–937)	1011 (838–1073)	879 (842–899)	1066 (875–1220)	1073 (816–1087)	860 (824–911)
	ELWI (ml/kg)	10 (10–13)	8 (8–8)	10 (10–11)	9 (9–9)	11 (10–16)	8 (8–9)	15 (11–22)	9 (9–11)*	11 (10–11)	12 (12–12)
	PVPI	2.4 (2.1–3.0)	2.1 (2.0–2.1)	2.7 (2.6–3.0)	2.5 (2.5–2.8)	3.1 (2.9–3.6)	1.8 (1.6–2.2)	3.7 (3.2–5.1)	2.0 (1.9–2.2)*	2.2 (2.0–3.0)	2.9 (2.9–3.1)
	CPI (W/m^2^)	0.80 (0.77–0.86)	0.66 (0.64–0.90)	0.89 (0.79–0.95)	0.75 (0.66–0.92)	0.59 (0.51–0.81)	0.77 (0.76–0.79)	0.41 (0.35–0.72)	0.83 (0.71–1.08)*	1.03 (0.65–1.39)	1.02 (0.91–1.18)
Respiratory system	Arterial pCO_2_ (mmHg)	41 (38–45)	42 (41–42)	48 (45–48)	44 (44–44)	51 (46–52)	45 (44–48)	61 (61–63)	50 (50–52)	47 (42–50)	56 (40–58)
	PaO_2_ (mmHg)	100 (99–102)	128 (112–129)	117 (108–129)	106 (101–109)	94 (85–97)	85 (84–88)	74 (63–96)	83 (79–87)	68 (64–88)	71 (69–75)
	FiO_2_ (%)	31 (28–34)	31 (30–32)	35 (34–37)	32 (30–34)	41 (39–50)	34 (34–40)	82 (81–87)	49 (44–50)*	51 (47–64)	56 (48–64)
	PaO_2_/FiO_2_ (mmHg)	332 (312–346)	375 (359–412)	329 (303–348)	314 (303–321)	193 (164–223)	251 (209–266)	105 (77–128)	174 (170–180)	136 (100–172)	130 (118–145)
	Respiratory rate	17 (17–18)	14 (14–16)	20 (19–20)	16 (16–18)	25 (23–27)	18 (17–20)	35 (32–35)	20 (19–22)*	23 (23–27)	25 (21–28)
	PEEP (mbar)	1 (1–1)	1 (1–1)	3 (2–4)	0 (0–1)	4 (2–7)	2 (1–4)	15 (14–17)	1 (1–5)*	2 (0–3)	5 (2–7)
	Inspiratory plateau pressure (cmH_2_O)	18 (15–19)	15 (15–16)	20 (18–21)	18 (18–20)	25 (25–30)	22 (21–28)	34 (33–36)	27 (25–30)	27 (27–28)	32 (31–33)
	SPO_2_ (%)	99 (98–99)	99 (98–99)	98 (98–99)	98 (98–99)	98 (94–98)	97 (97–97)	92 (76–97)	97 (93–98)	92 (87–95)	91 (89–92)
	End tidal CO_2_ (mmHg)	43 (41–45)	42 (41–44)	45 (44–47)	42 (42–44)	45 (43–47)	43 (41–45)	42 (12–46)	44 (40–46)	41 (35–42)	47 (42–49)
	Tidal volume (ml)	510 (495–525)	500 (500–500)	505 (495–513)	500 (500–500)	465 (460–478)	490 (480–500)	380 (368–413)	480 (440–480)	460 (440–480)	460 (428–485)
	Blood pH	7.53 (7.50–7.55)	7.49 (7.45–7.50)	7.46 (7.42–7.48)	7.42 (7.41–7.43)	7.39 (7.37–7.43)	7.42 (7.39–7.43)	7.28 (7.21–7.34)	7.39 (7.39–7.40)*	7.44 (7.38–7.44)	7.35 (7.33–7.43)
	Arterial HCO_3_^−^ (mmol/l)	32.5 (30.8–34.3)	30.6 (28.4–32.3)	31.4 (31.2–33.1)	27.8 (27.3–28.6)	29.8 (28.6–30.7)	28.1 (27.9–28.2)	27.6 (27.4–30.6)	30.5 (29.5–30.5)	29.0 (28.2–29.3)	28.2 (27.4–28.7)
	Base excess (mmol/l)	9.4 (6.9–9.5)	6.7 (4.0–7.4)	6.6 (5.8–8.8)	2.9 (2.3–3.8)	3.9 (1.1–6.1)	2.9 (2.3–3.0)	−0.4 (−3.0 to 5.3)	4.3 (3.7–4.6)	3.6 (3.5–3.8)	1.9 (1.2–3.3)
CNS	ICP (mmHg)	11.3 (8.2–11.5)	11.6 (9.6–12.5)	12.4 (12.4–15.6)	12.2 (10.7–13.5)	18.1 (15.4–20.9)	14.9 (13.0–16.1)	15.8 (13.4–17.8)	14.9 (12.1–16.1)	11.3 (11.0–14.7)	15.8 (14.0–17.4)
	CPP (mmHg)	55.6 (51.0–57.5)	57.0 (53.0–70.0)	48.0 (41.0–52.0)	56.0 (55.4–58.0)	43.2 (36.0–44.0)	46.0 (45.0–47.0)	21.0 (−0.8 to 28.0)	47.0 (41.0–50.0)*	55.0 (47.0–58.0)	52.5 (45.6–59.5)
	Intracranial temperature (°C)	38.1 (37.2–38.1)	37.7 (37.4–38.0)	38.0 (37.7–38.8)	37.9 (37.8–38.3)	38.3 (37.5–38.6)	37.4 (37.5–38.2)	38.7 (37.6–38.0)	37.5 (37.5–38.2)	37.6 (37.6–38.0)	38.3 (38.0–38.7)
Liver	Total bilirubin (mg/dl)	5.1 (4.1–5.2)	4.7 (4.0–5.7)	5.3 (4.5–6.0)	4.8 (4.8–6.0)	5.2 (4.9–5.6)	2.7 (2.4–3.4)	5.5 (4.6–5.6)	2.3 (2.3–3.0)*	2.2 (2.0–3.3)	2.5 (2.5–3.5)
	Lactate (mmol/l)	1.5 (1.4–1.6)	1.1 (0.9–1.7)	2.3 (2.0–2.4)	2.1 (2.0–2.8)	4.8 (3.7–5.6)	3.7 (3.5–3.9)	8.3 (7.9–8.6)	4.2 (3.5–4.2)*	5.4 (4.0–5.7)	5.8 (3.9–7.4)
	Ammonia (μg/dl)	264 (239–420)	167 (148–215)	305 (191–595)	152 (139–206)	374 (292–555)	161 (148–188)	681 (595–851)	194 (187–196)*	187 (167–228)	255 (170–313)
	Alkaline phosphatase (U/l)	228 (209–236)	246 (235–254)	280 (247–334)	274 (252–298)	322 (276–393)	262 (237–271)	307 (260–393)	230 (223–266)	251 (234–266)	253 (244–277)
	GPT (U/l)	52 (34–58)	46 (36–47)	46 (31–53)	43 (33–44)	41 (36–47)	34 (28–35)	35 (21–39)	31 (30–33)	33 (26–34)	35 (29–37)
	GOT (U/l)	98 (75–144)	56 (48–57)	75 (62–167)	45 (42–51)	85 (67–1449	39 (38–49)	97 (75–101)	51 (49–70)*	72 (67–97)	104 (86–113)
	LDH (U/l)	672 (564–871)	597 (518–611)	559 (482–735)	518 (457–563)	524 (473–1051)	371 (358–486)	743 (601–795)	420 (401–548)*	503 (502–663)	642 (631–719)
	Total protein (g/dl)	5.5 (5.4–5.7)	5.5 (5.4–5.7)	5.1 (4.9–5.4)	5.3 (5.2–5.4)	4.6 (4.5–4.7)	4.4 (4.3–4.4)	4.1 (4.1–4.4)	4.0 (4.0–4.4)	4.2 (4.1–4.2)	4.1 (4.0–4.1)
Kidney	Creatinine (mg/dl)	1.4 (1.4–1.5)	1.6 (1.4–1.7)	1.4 (1.4–1.5)	1.5 (1.5–1.6)	2.0 (1.7–3.0)	1.3 (1.2–1.7)	2.3 (2.1–3.3)	1.4 (1.1–1.7)*	1.4 (1.1–1.8)	1.7 (1.3–1.9)
	BUN (mg/dl)	15 (14–15)	11 (9–12)	16 (14–17)	12 (10–13)	16 (13–23)	8 (6–11)	17 (14–20)	6 (5–9)*	5 (4–7)	5 (4–6)
	Infused fluids (ml/h)	286 (285–326)	562 (403–604)	1116 (510–1407)	479 (355–700)	970 (295–1702)	553 (463–910)	385 (310–457)	441 (422–472)	375 (331–388)	265 (155–370)
	Urine output (ml/h)	100 (34–150)	130 (110–170)	64 (50–100)	65 (60–75)	40 (40–45)	50 (40–60)	35 (22–40)	60 (45–65)*	50 (36–50)	50 (40–50)
Coagulation and hematology	Quick value (%)	103 (88–112)	87 (86–88)	107 (105–109)	100 (93–102)	98 (88–103)	82 (77–85)	84 (70–91)	83 (72–86)	82 (76–85)	79 (71–79)
	INR	1.0 (0.9–1.1)	1.1 (1.1–1.1)	1.0 (0.9–1.0)	1.0 (1.0–1.0)	1.1 (1.0–1.1)	1.1 (1.1–1.2)	1.1 (1.1–1.2)	1.1 (1.1–1.2)	1.1 (1.1–1.2)	1.2 (1.2–1.2)
	Fibrinogen (mg/dl)	662 (534–794)	725 (668–756)	623 (615–708)	663 (651–681)	479 (414–532)	579 (553–596)	533 (492–594)	558 (502–572)	506 (456–574)	540 (446–612)
	Platelet count (10^3^/μl)	504 (333–541)	367 (356–428)	216 (195–286)	234 (226–308)	140 (125–141)	170 (134–180)	131 (30–138)	144 (93–150)	116 (85–143)	113 (83–133)
	Hemoglobin (g/dl)	10.5 (10.3–10.8)	9.9 (9.9–11.1)	11.6 (11.4–12.1)	12.2 (11.7–12.6)	12.0 (11.4–12.2)	10.8 (10.5–11.0)	12.9 (12.4–12.9)	10.5 (9.8–11.7)	10.4 (10.3–11.7)	11.5 (10.7–11.8)
	Hematocrit (%)	33 (32–35)	33 (32–37)	38 (37–39)	40 (38–42)	39 (37–40)	36 (34–39)	43 (42–46)	36 (33–41)	35 (34–39)	38 (36–39)
	White blood cells (10^3^/μl)	15.2 (14.5–17.6)	18.3 (18.2–20.1)	3.0 (2.8–3.1)	3.1 (2.6–3.1)	1.0 (1.0–1.0)	1.2 (1.0–1.4)	1.9 (0.8–2.0)	0.7 (0.7–0.8)	1.3 (1.1–1.4)	3.1 (2.8–3.8)
Laboratory values	Na^+^ (mmol/l)	138 (138–139)	139 (139–140)	139 (138–142)	138 (138–139)	138 (138–140)	138 (137–140)	138 (137–139)	140 (138–140)	139 (138–141)	138 (137–139)
	K^+^ (mmol/l)	3.7 (3.5–3.9)	4.5 (4.0–4.5)	3.7 (3.4–3.7)	3.9 (3.7–4.2)	3.5 (3.4–4.0)	3.3 (3.3–3.5)	4.5 (4.1–4.8)	3.6 (3.5–3.9)*	3.7 (3.6–3.7)	4.5 (4.2–4.6)
	Total calcium (mg/dl)	2.53 (2.39–2.62)	2.62 (2.59–2.73)	2.4 (2.35–2.41)	2.57 (2.54–2.70)	2.44 (2.35–2.54)	2.47 (2.42–2.50)	2.38 (2.32–2.54)	2.28 (2.27–2.40)	2.30 (2.28–2.37)	2.23 (2.13–2.31)
	Ionized calcium (mmol/l)	1.27 (1.24–1.31)	1.40 (1.30–1.41)	1.32 (1.27–1.33)	1.42 (1.32–1.43)	1.31 (1.30–1.35)	1.36 (1.36–1.39)	1.29 (1.26–1.31)	1.31 (1.30–1.33)	1.31 (1.26–1.34)	1.24 (1.18–1.26)
	Chloride ion (mmol/l)	102 (99–102)	104 (100–104)	101 (100–102)	104 (101–104)	101 (99–102)	104 (102–104)	101 (99–101)	104 (103–105)	103 (102–105)	102 (101–103
	Anion gap (mmol/l)	11.6 (9.0–11.9)	9.2 (8.8–13.0)	10.6 (10.1–12.8)	12.0 (11.7–12.1)	12.5 (11.4–13.3)	10.3 (9.3–11.6)	15.8 (15.6–16.3)	9.9 (8.5–10.3)	10.4 (9.7–12.6)	12.7 (9.1–14.3)
	Glucose (mg/dl)	100 (98–104)	105 (105–110)	106 (97–115)	103 (100–109)	138 (130–151)	122 (120–142)	153 (137–212)	129 (119–135)	126 (106–138)	138 (123–161)
	Phosphate (mg/dl)	8.2 (8.0–9.9)	8.2 (7.5–8.7)	9.2 (7.9–9.8)	8.4 (7.9–9.1)	8.6 (7.7–9.4)	7.2 (6.8–7.3)	8.6 (8.1–10.5)	7.1 (6.6–7.5)*	7.5 (6.7–7.6)	8.1 (7.7–8.3)
	Magnesium (mmol/l)	0.93 (0.80–0.93)	0.82 (0.78–0.89)	0.82 (0.79–0.89)	0.77 (0.75–0.84)	0.81 (0.79–0.83)	0.84 (0.81–0.92)	0.96 (0.89–0.99)	0.92 (0.86–0.96)	0.99 (0.88–1.00)	1.00 (0.91–1.01)
	Osmolality (mosmol/kg)	289 (289–292)	292 (289–293)	290 (288–293)	290 (288–290)	291 (289–292)	292 (286–293)	298 (295–301)	293 (287–294)	293 (292–293)	295 (290–295)
	Albumin (g/dl)	2.7 (2.7–2.8)	2.7 (2.6–2.9)	2.5 (2.5–2.5)	2.6 (2.4–2.6)	2.2 (2.1–2.3)	2.1 (2.0–2.2)	2.0 (1.8–2.1)	1.9 (1.9–2.0)	2.0 (2.0–2.0)	1.9 (1.7–2.1)
	Alpha1-globulin (g/dl)	1.1 (0.9–1.1)	1.1 (1.0–1.1)	1.0 (1.0–1.0)	1.0 (1.0–1.0)	0.9 (0.8–1.0)	0.8 (0.8–0.9)	0.9 (0.8–1.0)	0.7 (0.7–0.9)	0.8 (0.8–0.8)	0.8 (0.7–0.8)
	Alpha2-globulin (g/dl)	0.3 (0.3–0.3)	0.4 (0.3–0.4)	0.2 (0.2–0.3)	0.3 (0.2–0.4)	0.3 (0.2–0.3)	0.2 (0.2–0.3)	0.2 (0.2–0.2)	0.2 (0.2–0.2)	0.2 (0.2–0.2)	0.2 (0.2–0.2)
	Beta-globulin (g/dl)	0.6 (0.6–0.7)	0.6 (0.6–0.7)	0.6 (0.6–0.7)	0.6 (0.5–0.7)	0.6 (0.5–0.5)	0.5 (0.5–0.6)	0.5 (0.5–0.5)	0.5 (0.5–0.5)	0.5 (0.5–0.6)	0.5 (0.5–0.5)
	Gamma-globulin (g/dl)	0.8 (0.7–0.9)	0.9 (0.9–0.9)	0.8 (0.6–0.8)	0.8 (0.8–0.8)	0.7 (0.6–0.8)	0.7 (0.7–0.7)	0.6 (0.5–0.7)	0.6 (0.6–0.7)	0.7 (0.6–0.7)	0.6 (0.6–0.7)
Anesthesia	Propofol infusion (ml/h)	38 (30–40)	45 (40–50)	38 (35–45)	45 (38–52)	40 (32–42)	35 (35–45)	38 (30–45)	32 (20–45)	35 (28–35)	38 (35–40)
	Remifentanil infusion (ml/h)	0.5 (0.5–0.5)	0.2 (0.2–0.3)	0.3 (0.2–0.3)	0.2 (0.2–0.3)	0.3 (0.2–0.3)	0.3 (0.2–0.3)	0.2 (0.2–0.2)	0.2 (0.1–0.2)	0.2 (0.1–0.2)	0.2 (0.2–0.3)

*CNS* central nervous system, *MAP* mean arterial pressure, *CVP* central venous pressure, *SVRI* systemic vascular resistance index, *GEDI* global end-diastolic volume index, *ITBI* intrathoracic blood volume index, *ELWI* extravascular lung water index, *PVPI* pulmonary vascular permeability index, *CPI* cardiac power index, *PaO*_*2*_ arterial oxygen pressure, *FiO*_*2*_ fraction of inspiratory oxygen, *PEEP* positive end-expiratory pressure, *SpO*_*2*_ peripheral oxygen saturation, *ICP* intracranial pressure, *CPP* cerebral perfusion pressure, *GPT* glutamate-pyruvate transaminase, *GOT* glutamic oxaloacetic transaminase, *LDH* lactate dehydrogenase, *BUN* blood urea nitrogen, *INR* international normalized ratio

*p < 0.05 (control vs. ADVOS)

^a^The statistical analysis to analyze differences between control and ADVOS groups was performed with the last available values of the whole study set at the closest time point to T6 (*n* = 10)

### Survival

**Fig. 3:**
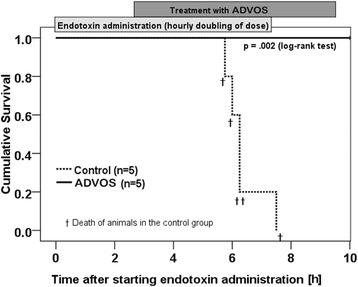
Log-rank comparison of survival rates between control and ADVOS group systems from T0 to T10 after endotoxin administration. Labels for ADVOS and endotoxin administration are placed in a way that shows the duration of each procedure. All animals in the control group died, while animals treated with ADVOS survived the whole observation period

All animals in the control group died within 7½ h of starting endotoxemia (Fig. 3). On the contrary, animals in the ADVOS group survived the 10-h observation period (*p* = 0.002). Since most of the animals in the control group died even before receiving the planned endotoxin dose (according to the dosing protocol), the mean total endotoxin dose was approximately one third (252 ± 128 μg/kg) of the full dose received by the ADVOS group (764 μg/kg).

### Effects of the ADVOS treatment in SOFA-related organ systems

#### Cardiovascular system

**Fig. 4:**
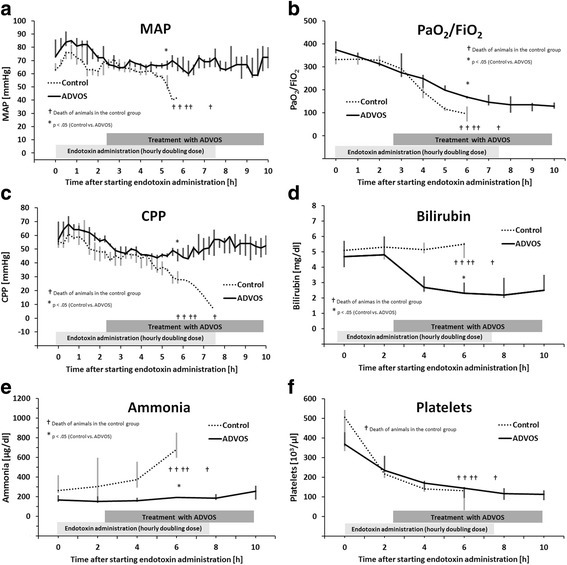
Effects of the ADVOS in treatment in SOFA score related surrogate markers from T0 to T10 after endotoxin administration. Each graphic shows the median and IQR. Labels for ADVOS treatment and endotoxin administration are placed in a way that shows the duration of each procedure. **a** Mean arterial pressure. **b** Ratio of oxygen arterial oxygen/fraction of inspired oxygen. **c** Cerebral perfusion. **d** Bilirubin. **e** Ammonia. **f** Platelet number

The ADVOS group showed a stable and significantly higher mean arterial pressure (MAP) than the control group at T6 (Fig. 4a). Diastolic blood pressure (36 vs. 44 mmHg) was also significantly different in the control and ADVOS group at T6, respectively. Significant differences were also found in different dynamic parameters such as the extravascular lung water index (ELWI) (15 vs. 9 ml/kg), the pulmonary vascular permeability index (PVPI) (3.7 vs. 2.0), the cardiac index (4.9 vs. 6.7 ml/min/m^2^), and the cardiac power index (CPI) (0.41 vs. 0.83 W/m^2^). No statistically significant differences were observed for central venous pressure (CVP), global end-diastolic volume index (GEDI), systemic vascular resistance index (SVRI), and systolic pressure (Table 2).

#### Respiratory system

Although PaO_2_ was similar among animals in both groups (74 mmHg in control vs. 83 mmHg in ADVOS, at T6), the needed FiO_2_ to keep sufficient oxygenation in the ADVOS group was significantly lower than that in the control group (82 vs. 49%, at T6), resulting in an improved PaO_2_/FiO_2_ ratio (105 vs. 174, at T6, Fig. 4b). The partial pressure of carbon dioxide (pCO_2_) at T6 was also significantly different (61 vs. 50 mmHg). To overcome hypoxemia and prevent higher hypercapnia, animals in the control group needed ventilation with higher respiratory rates (35 vs. 20) and PEEP (15 (IQR 14–17) vs. 1 (IQR 1–5) mbar).

Therefore, the animals in the control group developed severe respiratory failure associated with respiratory acidosis (pH 7.28, at T6), which was overcome in the ADVOS group (pH 7.39, at T6).

#### Central nervous system

Intracranial pressure was similar in both groups. However, the ADVOS group was able to maintain a stable higher CPP until the end of the observation period (Fig. 4c). The intracranial temperature was not different between control and treatment group (Table 2).

#### Liver

In comparison with the control group, animals in the ADVOS group had significantly lower concentration of bilirubin (5.5 vs. 2.3 mg/dl, at T6) (Fig. 4d). In addition, at T6, the ADVOS treatment was able to obtain reduced levels of ammonia (681 vs. 194 μg/dl) (Fig. 4e) and lactate (8.3 vs. 4.2 mmol/l). Specific enzymes such as alkaline phosphatase (ALP) and glutamate-pyruvate transaminase (GPT) were similar in both groups at T6, while glutamic oxaloacetic transaminase (GOT) and lactate dehydrogenase (LDH) were significantly lower in the ADVOS group (Table 2).

#### Kidney

Similarly, in the ADVOS group, reduced creatinine and blood urea nitrogen (BUN) levels were found throughout the study, in comparison with the control group (17 vs. 6 mg/dl, at T6, Table 2). Urine output was not significantly different between the two groups, and it was maintained throughout the 10-h observation period in the ADVOS group (Table 2).

#### Hemostasis and coagulopathy

No adverse events due to bleeding were documented. Endotoxin administration resulted in severe leukopenia and thrombocytopenia (Fig. 4f). The amount of platelets (131 vs. 144 10^3^/μl, at T6) and the international normalized ratio (INR) (1.1 in both cases) were similar in the control and the ADVOS group, respectively.

#### Laboratory values

As shown in Table 2, no significant differences were observed for sodium, potassium, total and ionized calcium, chloride, anion gap, glucose, phosphate, magnesium, osmolality, and albumin values.

#### Anesthesia

Propofol and remifentanil infusion rates were similar in both groups (Table 2).

#### Pathology

Pathological examination of animal liver postmortem showed early changes of the portal tract as a result of cholestasis. These changes included neutrophilic infiltration, biliary cell proliferation, and inspissated bile within dilated bile ducts.

## Discussion

In the present study, an animal model was developed following the two-hit etiology for MOF proposed by Meakins [14]. The model encompassed two steps, starting with the induction of a cholestatic liver dysfunction (1st hit) resulting in cholangitis, which was followed by a superimposed endotoxemia (2nd hit). The rationale behind this is that cholestasis has been described to be present in 20% of the patients during their stay in the ICU, being the most common feature of liver dysfunction and being associated with increased morbidity and mortality in this hospital ward [5, 26, 27]. Indeed, pre-existing liver dysfunction plays a pivotal role as a risk factor for the progress of infection into sepsis [7].

Three days post-surgery, comparable groups of animals were obtained with elevated mean levels of bilirubin, ammonia, fibrinogen, leukocytes, and aminotransferases, confirming a liver dysfunction and an inflammatory process. In addition, the renal system was also affected, revealed by an alteration in creatinine. This renal impairment at this point, adds a grade of severity to the model and highlights the multiple organ implication. Kidneys play a major role in ammonia clearance [28], and renal involvement worsens liver failure in 30–50% of the patients [29], which is associated with a poor prognosis once renal failure develops [30].

With further administration of endotoxins in an hourly twofold stepwise increments protocol, we were able to induce a septic shock in the pigs within 2 h after endotoxemia. The animals developed a capillary leak syndrome, thrombocytopenia, leukopenia, and deterioration of respiratory function. The administration of endotoxins at relatively low doses with gradual increments results in deterioration of respiratory function as part of the multiple organ failure, while the injection of a single high dose bolus is more likely to cause early death from pulmonary artery hypertension [31].

During the whole study and, particularly, during the treatment phase (T0 to T10), special attention was put to avoid biases between groups and more than 25 standard operating procedures were followed. In this regard, invasive hemodynamic monitoring based on transpulmonary thermodilution and pulse contour analysis has been shown to be useful to guide and monitor extracorporeal organ support [32, 33]. Therefore, we adjusted fluid therapy in both groups according to PiCCO parameters [10]. Similarly, acid-base and electrolyte levels, especially hyper- and hypokalemia, were also controlled by additional infusions [10]. Moreover, by definition, septic shock involves the administration of vasopressors in order to control the low MAP [34]. As done in our previous trial [10], this was avoided to prevent the addition of a confounding factor that could affect the proper interpretation of the results. Despite the lack of use of vasopressors, we provided standard critical care to the animals in many other aspects, i.e., in an ICU-like environment.

As previously shown for an acute liver failure (ALF) model [10], also in this different animal model including endotoxemia and further dysfunction of multiple organs, the ADVOS procedure prevented death in treated animals. In the previous model, we induced ischemic liver injury (by ligating hepatic artery and diverting portal venous blood through functional end-to-side portosystemic shunt). In contrast, in the present model, we performed portal vein diversion and ligation of major biliary ducts, without interrupting arterial supply, and then followed by endotoxemia. The latter step constitutes a major difference between the two models and, in our opinion, accelerated the inflammatory process. Some of the lab values were also different (e.g., fibrinogen). Our results on survival improvement are supported by the efficacy of the ADVOS procedure in eliminating protein-bound and water-soluble organ dysfunction markers [10] like ammonia. In the liver and the kidney, the ADVOS procedure replaces only the detoxification function and thereby lacks other functions, e.g., synthesis or hormonal regulation. We speculate that efficient detoxification lead to the overall improvement of organ function. In this line, results may be interpreted as progress for SOFA-related systems (cardiovascular, cerebral, renal, respiratory, and hepatic systems). In comparison to the control animals, the ADVOS group was able to maintain surrogate markers such as MAP, creatinine, PaO_2_/FiO_2_, and bilirubin at significantly better values. However, the reduction of serum creatinine and BUN is rather a function of dialysis and may not reflect improvement in renal function. In addition, taking into account that Glasgow Coma Scale (GCS) was not measurable in the animals, CPP levels were significantly higher in pigs treated with ADVOS, which has been shown to positively correlate with GCS [35].

Moreover, the improvement of tissue perfusion and tissue oxygenation through adequate cardiovascular function helped in the stabilization of blood pH and allowed more protective airway pressures. During the observation period, both groups of animals developed high-output heart failure as shown by an increased cardiac index and a reduced vascular resistance [36]. Animals in the ADVOS group were able to longer survive due to the improvement of cardiac performance, highlighted by more than double cardiac power index at T6 (0.41 vs. 0.83 W/m^2^). Even if an improvement in cardiac performance was observed, the direct pathogenic link between the removal of disease markers by the ADVOS procedure and the increase in the cardiac index still needs to be investigated. The improvement of the cardiac index could be a result of several mechanisms: (i) The significantly higher PEEP values due to decreased oxygenation in the control group could have lowered the cardiac output. (ii) As indicated by a higher capillary leak, a lower preload could have contributed to a decreased cardiac output. The infusion rate in the control group was limited by a higher ELWI but might have also been too low for an optimal cardiac output. (iii) A possible higher pulmonary pressure due to the endotoxin-induced arterial vasoconstriction in the control group could have resulted in an increased afterload [37], even worsening the effect of the increased PEEP. However, animals in the ADVOS group received three times more endotoxin amount during the whole observation period, indicating that the effect of either endotoxins or other factors contributing to the vasoconstriction might have been positively influenced by the ADVOS treatment. (iv) Additionally, the higher CVP observed due to one of the above cited mechanisms could have also reduced the venous return and contributed to a lower preload, resulting in decreased blood pressure in the control group.

If any of the two well-known liver and kidney dysfunction markers (i.e., bilirubin and creatinine, respectively) are also markers for this detoxification function needs further investigation.

Furthermore, no differences were shown in coagulation parameters between both groups. The number of platelets remained above 100,000/μl during the whole study, which reflects the lack of influence of the ADVOS system on platelet count.

This study might be limited by a small sample size and the restrictions of the animal model in terms of life expectancy. The early start of the ADVOS procedure (which was necessary due to the short lifetime of the animals and the expedited nature of events in the model) is indeed a disadvantage that may restrict extrapolation to critically ill patients. Nonetheless, our results refer to a promising system that may improve survival in different groups of patients requiring intensive care. So far, the main target groups for the application of liver support devices (e.g., MARS and Prometheus™) were patients with acute or with acute-on-chronic liver failure [38–40].

Even if good evidence has been provided, one should be cautious in view of the fact that preclinical data for many other sepsis models does not always correlate with similar outcomes in clinical trials [41]. In fact, different extracorporeal procedures and endotoxin adsorbers have been shown to improve endotoxin-induced organ failure and hypoxemia in different animal models [42–44], but no breakthrough treatment was observed in the last years. In contrast, compared to other devices, the ADVOS procedure provides several additional advantages such as a stable blood purification during the whole treatment and not only during the first 2 h [45, 46], a lower use of albumin (2 vs. 20% with MARS) with the corresponding decrease of the costs, and higher flow rates of dialysate (up to 60 l/h) in comparison to those routinely used by MARS (200 ml/min) or single pass albumin dialysis (1 l/h) [47, 48].

Considering these advantages, it would be of great help to directly compare the ADVOS system with other devices in animal studies. However, the lack of a standardized animal model and guidelines for its handling continues hampering the development of effective treatments.

## Conclusions

In the present work, we have developed a swine model with a sepsis-like syndrome with dysfunction of multiple organs consisting of two phases: induction of cholestatic liver injury and endotoxin administration. This model allowed us to analyze the safety and efficacy of the ADVOS procedure, resulting in an improvement of the survival rates; a decrease of bilirubin and creatinine levels; an improvement of the cardiovascular, respiratory, and central nervous system parameters; and a safe profile demonstrated by the absence of any treatment-related coagulation problems.

## Additional file

Additional file 1: Table S1. Pre-set FiO_2_/PEEP employed to maintain an adequate ventilation of the animals throughout the study. **Table S2.** Block randomization for animal inclusion into study group control or ADVOS. (DOCX 56 kb)

## Abbreviations

ADVOS: ADVanced Organ Support
ALF: Acute liver failure
ALP: Alkaline phosphatase
BUN: Blood urea nitrogen
CPI: Cardiac power index
CPP: Cerebral perfusion pressure
CVP: Central venous pressure
ELWI: Extravascular lung water index
FiO_2_: Fraction of inspiratory oxygen
GCS: Glasgow Coma Scale
GEDI: Global end-diastolic volume index
GLP: Good laboratory practice
GOT: Glutamic oxaloacetic transaminase
GPT: Glutamate-pyruvate transaminase
ICP: Intracranial pressure
INR: International normalized ratio
ITBI: Intrathoracic blood volume index
LDH: Lactate dehydrogenase
MAP: Mean arterial pressure
MOF: Multiple organ failure
PaO_2_: Arterial oxygen pressure
pCO_2_: Partial pressure of carbon dioxide
PEEP: Positive end-expiratory pressure
PVPI: Pulmonary vascular permeability index
SOFA: Sepsis-related organ failure assessment
SOPs: Standard operating procedures
SVRI: Systemic vascular resistance index
ZPF: Center for Preclinical Research
