# Supplementary material for: Evaluation of an ADVanced Organ Support (ADVOS) system in a two-hit porcine model of liver failure plus endotoxemia
Source: Intensive Care Med Exp. 2017 Jul 4;5:31. doi: 10.1186/s40635-017-0144-3 (PMC5496922; doi:10.1186/s40635-017-0144-3)
Supplement: Additional file 1: Table S1. — Pre-set FiO2/PEEP employed to maintain an adequate ventilation of the animals throughout the study. Table S2. Block randomization for animal inclusion into study group control or ADVOS. (DOCX 56 kb) [file 40635_2017_144_MOESM1_ESM.docx]

**Additional file 1**

Table S1. Pre-set FiO_2_/ PEEP employed to maintain an adequate ventilation of the animals throughout the study.

| **FiO_2_ [%]** | <0.4 | 0.4 | 0.5 | 0.6 | 0.7 | 0.8 | 0.9 | 1.0 |
| --- | --- | --- | --- | --- | --- | --- | --- | --- |
| **PEEP [mbar]** | 1-5 | 5-8 | 8-10 | 10 | 10-14 | 14 | 14-18 | 18-22 |

FiO2: fraction of inspiratory oxygen; PEEP: positive end-expiratory pressure.

Table S2. Block randomization for animal inclusion into study group Control or ADVOS.

| Block | 1 | 1 | 1 | 1 | 1 | 2 | 2 | 2 | 2 | 2 |
| --- | --- | --- | --- | --- | --- | --- | --- | --- | --- | --- |
| Exp No. | 1 | 2 | 3 | 4 | 5 | 6 | 7 | 8 | 9 | 10 |
| Group | Control | Control | ADVOS | ADVOS | Control | ADVOS | ADVOS | Control | Control | ADVOS |
